# Optimization of smartphone psychotherapy for depression and anxiety among patients with cancer using the multiphase optimization strategy (MOST) framework and decentralized clinical trial system (SMartphone Intervention to LEssen depression/Anxiety and GAIN resilience: SMILE AGAIN project): a protocol for a randomized controlled trial

**DOI:** 10.1186/s13063-023-07307-y

**Published:** 2023-05-22

**Authors:** Megumi Uchida, Toshiaki A Furukawa, Takuhiro Yamaguchi, Fuminobu Imai, Kanae Momino, Fujika Katsuki, Naomi Sakurai, Tempei Miyaji, Masaru Horikoshi, Hiroji Iwata, Sadamoto Zenda, Tsuguo Iwatani, Asao Ogawa, Akira Inoue, Masakazu Abe, Tatsuya Toyama, Yosuke Uchitomi, Hiromichi Matsuoka, Hisashi Noma, Tatsuo Akechi

**Affiliations:** 1 Department of Psychiatry and Cognitive-Behavioral Medicine, Nagoya City University Graduate School of Medical Sciences, Mizuho-cho, Mizuho-ku, Nagoya, Aichi 467-8601 Japan; 2 Center for Palliative Care and Psycho-oncology, Nagoya City University Hospital, Nagoya, Japan; 3 Department of Health Promotion and Human Behavior, Graduate School of Medicine/School of Public Health, Kyoto University, Kyoto, Japan; 4 Division of Biostatistics, Tohoku University Graduate School of Medicine, Sendai, Japan; 5 Department of Nursing Administration and Management, Nagoya City University Graduate School of Nursing, Nagoya, Japan; 6 Department of Psychiatric and Mental Health Nursing, Nagoya City University Graduate School of Nursing, Nagoya, Japan; 7 Cancer Solutions, Tokyo, Japan; 8 Department of Clinical Trial Data Management, Tokyo University Graduate School of Medicine, Tokyo, Japan; 9 National Center for Cognitive Behavior Therapy and Research, National Center of Neurology and Psychiatry, Tokyo, Japan; 10 Department of Breast Oncology, Aichi Cancer Center, Nagoya, Japan; 11 Division of Radiation Oncology, National Cancer Center Hospital East, Chiba, Japan; 12 Department of Breast Surgery, National Cancer Center Hospital East, Chiba, Japan; 13 Department of Psycho-Oncology Service, National Cancer Center Hospital East, Kashiwa, Japan; 14 Department of Palliative Medicine, Tohoku University School of Medicine, Miyagi, Japan; 15 Division of Gynecologic Oncology, Shizuoka Cancer Center, Shizuoka, Japan; 16 Department of Gynecology, Hamamatsu University School of Medicine, Shizuoka, Japan; 17 Department of Breast Surgery, Nagoya City University Graduate School of Medical Sciences, Aichi, Japan; 18 Behavioral and Survivorship Research Group, Innovation Center for Supportive, Palliative and Psychosocial Care, National Cancer Center Hospital, Tokyo, Japan; 19 Center for Public Health Sciences, National Cancer Center, Tokyo, Japan; 20 Department of Psycho-Oncology, National Cancer Center Hospital, Tokyo, Japan; 21 Institute of Statistical Mathematics, Tokyo, Japan

**Keywords:** Smartphone psychotherapy, Depression, Anxiety, Cancer, Behavioral activation, Assertion training, Problem-solving therapy, Psychoeducation

## Abstract

**Background:**

Cancer patients experience various forms of psychological distress. Their distress, mainly in the form of depression and anxiety, leads to poor quality of life, increased medical spending due to frequent visits, and decrease in treatment adherence. It is estimated that 30–50% among them would require support from mental health professionals: in reality, much less actually receive such support partly due to a shortage of qualified professionals and also due to psychological barriers in seeking such help. The purpose of the present study is to develop the easily accessible and the most efficient and effective smartphone psychotherapy package to alleviate depression and anxiety in cancer patients.

**Methods:**

Based on the multiphase optimization strategy (MOST) framework, the SMartphone Intervention to LEssen depression/Anxiety and GAIN resilience project (SMILE-AGAIN project) is a parallel-group, multicenter, open, stratified block randomized, fully factorial trial with four experimental components: psychosocial education (PE), behavioral activation (BA), assertion training (AT), and problem-solving therapy (PS). The allocation sequences are maintained centrally. All participants receive PE and then are randomized to the presence/absence of the remaining three components. The primary outcome of this study is the Patient Health Questionnaire-9 (PHQ-9) total score, which will be administered as an electronic patient-reported outcome on the patients’ smartphones after 8 weeks. The protocol was approved by the Institutional Review Board of Nagoya City University on July 15, 2020 (ID: 46-20-0005). The randomized trial, which commenced in March 2021, is currently enrolling participants. The estimated end date for this study is March 2023.

**Discussion:**

The highly efficient experimental design will allow for the identification of the most effective components and the most efficient combinations among the four components of the smartphone psychotherapy package for cancer patients. Given that many cancer patients face significant psychological hurdles in seeing mental health professionals, easily accessible therapeutic interventions without hospital visits may offer benefits. If an effective combination of psychotherapy is determined in this study, it can be provided using smartphones to patients who cannot easily access hospitals or clinics.

**Trial registration:**

UMIN000041536, CTR. Registered on 1 November 2020 https://center6.umin.ac.jp/cgi-open-bin/ctr/ctr_view.cgi?recptno=R000047301.

**Supplementary Information:**

The online version contains supplementary material available at 10.1186/s13063-023-07307-y.

## Background

It is estimated that there were 19.3 million new cancer cases and 10 million cancer deaths [1] worldwide in 2020. Cancer patients experience various forms of psychological distress, with 30–50% of them recommended to receive support from mental health professionals [2]. In the surveillance of Japanese cancer patients’ day-to-day concerns, the most frequent concern was psychological distress (33%) [3]. Among the developed countries, Japan has an extremely high suicide rate, and cancer patients account for a major proportion of suicides in general hospitals [4]. Moreover, the suicide rate increases 24-fold within 1 year after cancer diagnosis [5]. In this context, it has been demonstrated that depression, rather than physical distress, contributes to suicide [6]. Depression and anxiety impact the quality of life [7] of cancer patients, increase medical spending, decrease treatment adherence [8], and elevate morbidity [9]. Therefore, depression and anxiety are found to impair many important outcomes in cancer care and are among the most frequent and greatest concerns for cancer patients and their families [10–12]. However, the evidence base for depression treatment focuses on a single cancer, particularly breast cancer. Considering the heterogeneity in cancer populations, research is required to investigate the efficacy of depression treatment among all the cancer population [13].

Although the number of cancer patients continues to increase, we have not been able to palliate psychological distress to a satisfactory extent. While cancer patients expect appropriate care and intervention for their depression and anxiety and the efficacy of pharmacotherapy and psychotherapy is well known, most patients do not receive appropriate treatment. The number of healthcare professionals who treat psychological distress is limited compared to the number of cancer patients. Patients have a significant psychological hurdle in seeing mental health professionals. Since cancer patients have many physical symptoms owing to their condition and treatment, healthcare professionals need to be aware of appropriate medications and their interactions with anticancer drugs, and a high degree of specialization is required during treatment. Among other hurdles, as most patients hope to receive psychological treatment rather than psychotropic drug therapy [14], easily accessible psychological interventions have become imperative.

The usability of smartphone-based cognitive behavioral therapy (CBT) for depression in general psychiatry settings was shown in a randomized controlled trial (RCT) [15]. However, this therapy includes multiple components, and its usability for cancer patients has not been demonstrated [16]. Considering that there are often communication barriers with important “others”, such as family and medical staff, and the use of assertion skills in clinical practice, we have added smartphone assertion training. For smartphone problem-solving therapy, our previous clinical trials with breast cancer survivors, without a control group, indicated preliminary usability [17]. We considered the above combination, estimating it to be the most appropriate by our clinical experience.

This study aims to establish a treatment method based on evidence and develop the most appropriate psychological intervention to alleviate the symptoms of depression and anxiety experienced by cancer patients. In relation to the component of psychotherapy based on these forms of CBT, we will conduct a fully factorial RCT for cancer patients to estimate the effect of each component and develop the most efficient and effective smartphone psychotherapy package to address their depression and anxiety.

This protocol is in accordance with the Standard Protocol Items: Recommendations for Interventional Trials guideline [18]. The present study is subject to the ethical guidelines for clinical studies published by the Japanese Ministry of Education, Science and Technology and the Ministry of Health, Labor and Welfare, and the modified Act on the Protection of Personal Information, as well as the ethical principles established for research with humans stipulated in the Declaration of Helsinki and further amendments thereto. The report is based on protocol version 1.1, approved on July 15, 2020, by the Nagoya City University Graduate School of Medical Sciences and the Nagoya City University Hospital Ethics Committee.

## Methods/design

### The aim

The aim of this study is to develop the most efficient and effective smartphone psychotherapy package to address depression and anxiety in cancer patients after diagnosis to improve their quality of life.

### Design

The multiphase optimization strategy (MOST) is an innovative approach based on engineering and behavioral sciences that provides a principled and comprehensive framework for selecting intervention components among multicomponent interventions [19, 20]. It consists of three stages: (1) preparation to conduct an optimization trial; (2) optimization to reveal what constitutes an optimized intervention; and (3) evaluation of the optimized intervention for an established intervention in an RCT. This study represents the optimization phase, in accordance with the MOST framework, and uses a fully factorial design to allow for the estimation of the main effects of individual components and their interaction effects.

This study is a parallel-group, multicenter, open, stratified block randomized, fully factorial trial with four experimental components: psychoeducation (PE), behavioral activation (BA), assertion training (AT), and problem-solving (PS). PE is provided to everyone but the three other components will be coded at two levels (presence and absence).

In this design, the effect of a component is estimated by comparing the mean of all combinations, including that component, against the mean of all other combinations that do not include the component. Since both the former and the latter sets of combinations have equal credibility and nonspecific supportive elements, this design allows for the estimation of effects specific to that component and can uniquely overcome the issue of specificity in psychotherapy research [21].

### Participants

#### Setting of the study

This trial will take place at Nagoya City University Hospital, Tohoku University Hospital, the National Cancer Center Hospital, the National Cancer Center Hospital East and Shizuoka Cancer Center in Japan. Collaborating hospitals/clinics will be recruited in a later stage of the study.

#### Eligibility criteria for study centers

Collaborating hospitals/clinics will be recruited from core facilities in areas with many cancer patients in Japan, taking into consideration their willingness to participate.

#### Eligibility criteria for participants

Each participant must satisfy all of the following inclusion criteria and none of the exclusion criteria.

Inclusion criteria are as follows:

- 1. Patients with a cancer diagnosis and awareness of the same diagnosis
- 2. Patients aged 20 years or older
- 3. Smartphone users who are capable of using an electronic patient-reported outcome (e-PRO) system with a smartphone
- 4. Patients with an estimated prognosis of more than 6 months
- 5. Patients who expect to concentrate on the intervention for about two months after participation in this study (e.g., those who do not plan to use cell-damaging anticancer drugs with strong side effects).

Exclusion criteria are as follows:

- 1. Patients who do not understand Japanese
- 2. Patients with psychiatric symptoms (e.g., dementia, cognitive impairment, and severe depression with suicide ideation) who have been identified by attending physicians as inappropriate for the study
- 3. Individuals who have received face-to-face CBT
- 4. Persons who received smartphone CBT or participated in our previous study, the “SMILE PROJECT.”

Eligibility criteria for study personnel are as follows:

Encouragement emails will be handled by personnel who are qualified in health care, such as nurses.

### Interventions

**Fig. 1:**
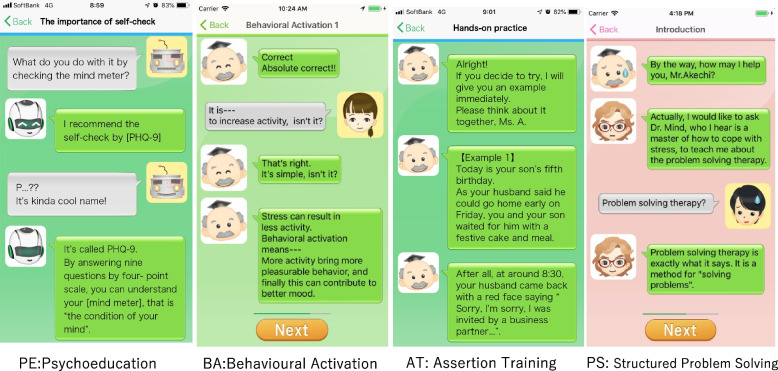
Smartphone psychotherapy. PE, psychoeducation; BA, behavioural activation; AT, assertion training; PS, structured problem solving

Figure 1 shows screenshots of the smartphone application. The components of smartphone psychotherapy are as follows:

PE consists of materials regarding psychological stress, emphasizing the importance of self-checks of one’s own emotional state. This is a basic matter and will be provided to all the participants.

- BA includes PE on the importance of pleasurable activities according to the principle “When your body moves, so does your mind.” It includes a worksheet for a personal experiment to test a new activity and a gamified “action marathon” to promote such personal experiments.
- AT consists of PE on assertive communication, in contrast to aggressive or passive communication. The participants will learn how to express their emotions and desires without hurting others or sacrificing their own pleasures.
- Structured problem solving (PS) teaches the participants how to evaluate the issues at hand, specify a concrete and achievable objective for the issue, conceptualize possible solutions, compare their advantages and disadvantages, and finally choose the most desirable action and perform it. A worksheet will be provided to guide the participants through this process.

Each component consists of a lesson that is supposed to take 1 week to complete. The participants can move to the next lesson only after 1 week has passed and after they have completed one worksheet.

**Fig. 2:**
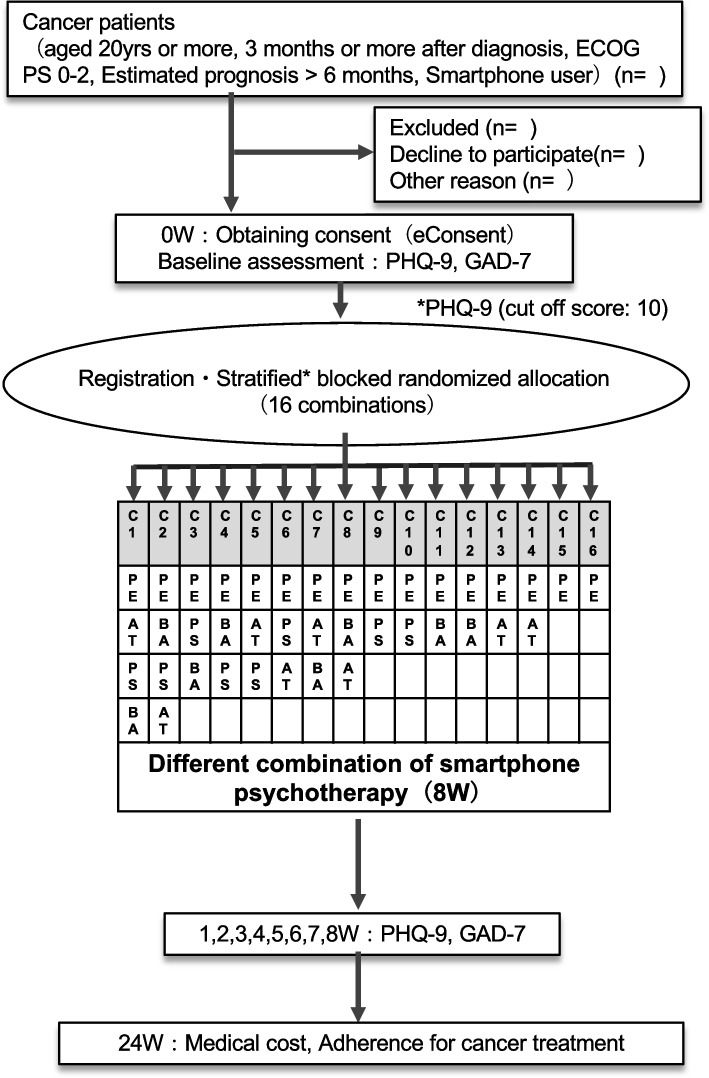
Flow, enrollment, intervention, and assessment schedule

PE constitutes the core element of the intervention, and all participants will receive this component. After completing PE, the participants will be randomly allocated to one of the combinations on the basis of the presence/absence of the remaining three components (Fig. 2).

To enable examination of the ordering effects between BA and PS, we doubled the number of combinations to 2^3^ × 2=16.

#### Strategies to improve adherence to interventions and procedures for monitoring adherence

The participants will receive a semiautomated email acknowledging their progress every week. The message is based on a template that focuses on adherence and motivation but can be modified by nurses on the study team. It can include technical advice for the program, but no therapeutic content with regard to CBT is allowed.

The participants’ adherence to the allocated intervention will be recorded and uploaded by the online server system. The study personnel will have access to the server and perform ongoing monitoring of the participants’ adherence.

#### Concomitant treatment

There is no restriction on concomitant treatments, except for specialized CBT.

#### Termination rules for participants

Discontinuation of protocol treatment

If a participant meets any of the following conditions, the research team can discontinue their access to the application. However, the participant will not be considered to have dropped out of the trial at that stage and will receive the protocol assessments if (1) the participant intends to stop the protocol treatment; (2) the research team judges that the risk of the protocol treatment is greater than the benefit for any reason; (3) the research team evaluates that it is difficult to continue the protocol treatment owing to clinical deterioration; and (4) the research team assesses that it is inappropriate to continue the protocol treatment for any reason (e.g., when identity theft, duplicate entry, etc., are detected).

## Anticipated risks and benefits for the participants

The intervention will consist of various amounts of PE and exercises in cognitive and behavioral skills for stress reduction conducted as self-help programs by the participants on their smartphone. The interventions will therefore be considered as “interventions with minimal invasiveness,” and no serious health risks are expected, except for possible psychological and time burdens while going through the program and responding to the questionnaires.

The possible psychological and time burdens will be fully disclosed and explained to the potential participants in video instructions and on the homepage, and only those who have provided e-consent will be recruited for this study. No insurance scheme is therefore planned.

However, the possibility of serious adverse events (SAEs), whether related to study participation or not, cannot be negated, as would normally be expected of the participants in their lives. All participants will be closely monitored, and they will be provided standard care in their hospitals. Any expenses necessary for such services will be handled as usual. On the other hand, participants can expect to learn about psychological stress and how to maintain mental health through the program. However, the extent of such benefits is the theme of this trial and is currently unknown.

## Description of materials

### Measurements

Table 1 shows the schedule for the outcome measurements.

**Table 1:**
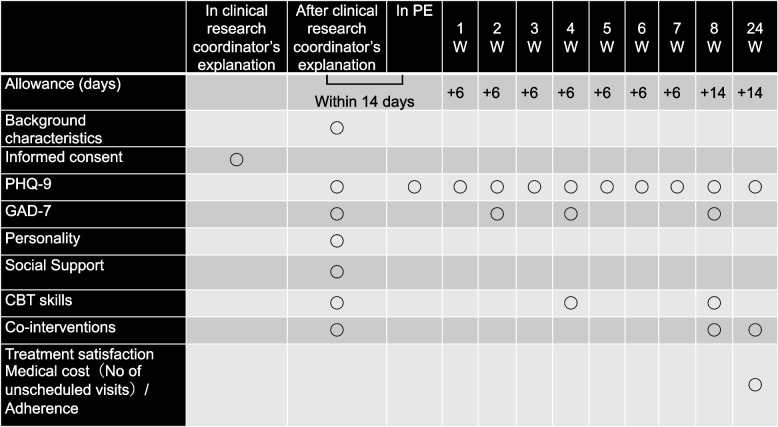
Schedule for the outcome measurements

### Primary outcome measure

The primary outcome is the self-administered Patient Health Questionnaire-9 (PHQ-9) [22] score at week 8.

### Secondary outcome measure

The secondary outcome is the self-administered Generalized Anxiety Disorder-7 (GAD-7) [23] score at week 8.

### Background characteristics

The following background characteristics will be measured at baseline.

- 1. Demographics
  a Age
  b Sex
  c Marital status (married/partnered, divorced/separated/bereaved, single)
  d Education (6, 9, 12, 14, 16, >16 years)
  e Employment status (full-time, part-time, home duties, unemployed)
  f Time since cancer diagnosis (>1 year, 1–3 years, 5–10 years, >10 years)
  g Original cancer site (breast, lung, stomach, colorectal, liver, uterus, ovary, prostate, head and neck, malignant lymphoma, other).
  h Cancer stage (0, I, II, III, IV, unknown, other) at cancer diagnosis
  i Current cancer stage (0, I, II, III, IV, unknown, other)
  j Previous cancer (Y/N)
  k Cancer treatment
    i Surgery (Y/N)
    ii Radiation therapy (Y (past, current)/N)
    iii Hormone therapy (Y (past, current)/N)
    iv Chemotherapy (Y (past, current)/N)
    v Targeted therapy (Y (past, current)/N)
    vi Immunotherapy (Y (past, current)/N)
- l. Eastern Cooperative Oncology Group Performance status (ECOG PS) (0, 1, 2)
- m. Hospital (Nagoya City University Hospital, Aichi Cancer Center, Tohoku University Hospital, the National Cancer Center Hospital, the National Cancer Center Hospital East, Shizuoka Cancer Center)
- n. Experience of consulting a doctor of psychiatry or psychosomatic medicine (past) (Y/N)
- o. Experience of consulting a doctor of psychiatry or psychosomatic medicine (current) (Y/N)
- p. Having tranquilizer or hypnotic drugs (Y/N)
- 2. Personality
  a Short form of the Big Five scale (29 items) [24]
- 3. Social support
  a Social Support Questionnaire (12 items) [25]
- 4. Cognitive and behavioral skills
  a Self-monitoring (five items) [26]
  b Cognitive restriction (six items) [27]
  c Behavioral activation: Behavioral Activation for Depression Scale-Short Form (eight items) [28]
  d Assertiveness (seven items) [29]
  e Problem solving (six items) [30]
- 5. Clinical characteristics
  a PHQ-9 [22] scores
  b GAD-7 [23] scores

### Patient health questionnaire-9 (PHQ-9) [22]

The PHQ-9 consists of nine diagnostic criteria items for an MDE in the *Diagnostic and Statistical Manual of Mental Disorders, Fourth Edition* (DSM-IV) [31] and the DSM-5 [32]. Each item is rated from 0 (*Not at all*) to 3 (*nearly every day*), with a total score ranging between 0 and 27. The reliability and validity of the original PHQ-9 and its Japanese version are well established [33, 34]. We changed the PHQ-9 recall time frame from 2 weeks to 1 week to measure weekly depression severity in this study [35].

## Generalized anxiety disorder-7 (GAD-7) [23]

The GAD-7 questionnaire was developed to measure the severity of generalized anxiety and includes seven items representing nervousness, tension, and worry. Each item is rated from 0 (*not at all*) to 3 (*nearly every day*), with the total score ranging between 0 and 21. Its reliability and validity have been established [23]. We changed the GAD-7 recall time frame from 2 weeks to 1 week to measure anxiety severity in this study [35].

## Big five scale of personality trait adjectives [36]

We will use the short form of the Big Five Scale of Personality Trait Adjectives, which is commonly used in Japan. The reliability and validity of the short version have been ascertained [24]. Each of the five personality traits of neuroticism, extraversion, openness, agreeableness, and conscientiousness will be measured with five to seven corresponding adjectives on a 5-point Likert scale from 0 (*Untrue of me)* and 4 (*True of me)*. We excluded the option “neither”, which is used in the original version [35].

## Social support questionnaire [25]

We will measure the size and quality of a patient’s social support using the short form of the Social Support Questionnaire. It measures the number of persons providing support and the satisfaction with such support in six domains. The reliability and validity of the original scale [25] and its Japanese version [37] have been satisfactory.

## Cognitive and behavioral skills

To measure each of the cognitive or behavioral skills for the three components in this trial, we will use the Short Cognitive Behavior Therapy Skills scale, whose reliability and validity have been established [38]. It measures a patient’s skills in BA, PS, and AT.

With regard to BA, we used the BA subscale of the Japanese Behavioral Activation for Depression Scale-Short Form [39], translated from the original English version [28], and validated it in the Japanese population. The Japanese version consists of five items, each rated from 0 (*very untrue) to* 3 (*very true).*

For AT, we will use the self-assertion subscale of the Adult Social Skills Scale, developed and validated by Aikawa et al. [29]. It comprises seven items, each rated between 0 *(very untrue of me)* and 3 *(very true of me).* Its reliability and validity have been established [29].

For PS, we will select the six highest loading items of the approach-avoidance style subscale of the Problem-Solving Inventory [30]. The reliability and validity of the original version have been established, and those of the Japanese version have been shown in another study. Each item is rated from 0 *(very untrue of me)* to 3 *(very true of me)*, and the total score ranges between 9 and 18.

### Procedure

#### Participant flow

Figure 1 shows the participant flow, enrollment, intervention, and assessment schedule.

#### Recruitment

Oncologists in the core cancer hospitals will introduce this research to subjects by giving them a card containing a QR code linked to the research homepage. Those who are interested in participating in the research will apply for participation from the homepage, and an email requesting participation will be sent to the secretariat. We will start sampling at Nagoya City University Hospital and then extend recruitment to the other core cancer hospitals in Japan. In the second year of the study, we will seek collaboration from several other core cancer hospitals/clinics in Japan.

#### Research managing system

**Fig. 3:**
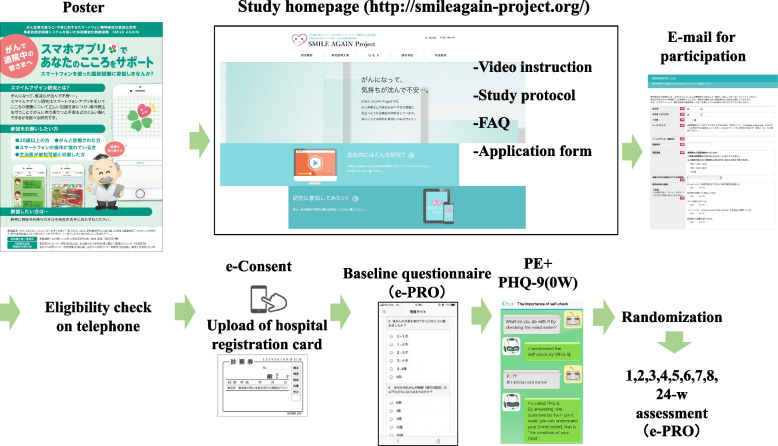
Study management system

This is a fully decentralized individually randomized, parallel-group multicenter trial that the subjects could participate into the trial without involving in-person contact which is similar to our previous study [40] (Fig. 3). The study’s website (http://smileagain-project.org/) provides further information.

The website explains the purpose of the study and the eligibility criteria and methods used; it also features a video that briefly introduces the study as well as provides full written information about it. The clinical research coordinators (CRCs) at the central office will ascertain their eligibility by telephone.

#### Electronic informed consent and randomization at week 0

After screening, the CRCs will seek to obtain the subjects’ electronic informed consent (e-consent) via the e-PRO system at week 0. Participants will be asked to upload a picture of their identification materials (patients will be especially encouraged to attach a photo of the ID card from the hospital where they made regular follow-up visits for cancer). This e-consent procedure is in accordance with the guidance of the US Food and Drug Administration. The original informed consent material is shown in the online Supplementary appendix.

After providing e-consent, completing the baseline investigation by the e-PRO system and and completing the PE component, the participants will be randomly allocated to one of the combinations using the electronic data capturing (EDC) web program at the data management center (Fig. 3). The random allocation will therefore be concealed.

If participants are allocated to the intervention group, which does not include all three components, they will be informed that the use of the remaining components can be resumed, if they wish, after week 24.

#### Trial period: weeks 0–8

The research nurses will send weekly emails for 8 weeks, encouraging the participants’ adherence to the application and asking them to record their responses on the e-PRO system. The research team can evaluate a patient’s progress using the application (e.g., the number of times and duration of use for each application) on the server.

#### Follow-up period: weeks 8–24

At week 24, the participants will receive an email encouraging them to provide their responses on the e-PRO system.

#### Termination assessment

If participants withdraw consent for assessment, they will not be followed up. Subjects will be excluded from the intention-to-treat (ITT) cohort of the trial if their characteristics are found to meet any exclusion criteria at baseline (e.g., aged under 20 years) after participation.

#### Assignment of interventions

Randomization and allocation concealment

The allocation will be stratified by the baseline PHQ-9 score (9 or less vs. 10 or more). We will use permuted block randomization to ensure balance in the number of subjects allocated to each combination because in the fully factorial design, imbalance in the allocated numbers among the factors would reduce the statistical power of the study. An independent data center will generate computer-generated random allocation sequences, which are maintained centrally, and the results of the assignment will determine which components are presented to the participants on their smartphone.

## Blinding

Neither the participants nor the study personnel will be blinded to the intervention that each participant is receiving through the conduct of the trial. The assessment of all the primary and secondary outcomes will be self-reported by the participants and therefore not blinded. The statisticians will be blinded to the allocation through the statistical analyses by analyzing the datasets prepared by the study personnel in which all components are denoted only by a letter.

### Plans to promote participant retention and complete follow-up

The outcome data will be collected even when a participant has not completed the allocated intervention unless he or she has expressed a wish to completely withdraw from the study. When a participant fails to fill in the scheduled assessments, reminders will be sent in 24 h and 48 h via an automated popup on the smartphone. If participants fail to provide their responses regarding the PHQ-9, a research nurse blinded to the assignment will send an e-mail to the subjects to elicit their answers. The participants will receive modest compensation for the time it requires to complete the questionnaire: 2000 yen when they complete the week 0 assessment, 1000 yen when they complete the week 4 assessment, 1000 yen when they complete the week 8 assessment, and 1000 yen for completing the week 24 assessment.

#### Data management

A secure, web-based, password-protected database will be used to manage recruitment, eligibility assessments, randomization, scheduling and tracking, baseline and follow-up assessments, and the delivery of the allocated interventions. All the assessment data will be checked automatically for integrity by this platform. The security of the data transfer between the application and the server will be guaranteed through the Secure Socket Layer (SSL).

### Statistical analysis and power calculation

#### Primary analyses

We will use the mixed-effects repeated-measures (MMRM) analysis [41] to estimate the mean difference in change scores on the PHQ-9 for each component. We will use the following regression model for the outcome variable of *i*th participant and *j*th visit ($i=\mathrm{1,2},\dots ,n;j=\mathrm{1,2},\dots ,T$),

$$E\left[{Y}_{ij}\right]={\beta }_{0}+{\beta }_{1}\times B{A}_{i}+{\beta }_{2}\times {AT}_{i}+{\beta }_{3}\times {PS}_{i}+{\beta }_{4}\times visi{t}_{j}+{\beta }_{5}\times ag{e}_{i}+{\beta }_{6}\times se{x}_{i}+{\beta }_{7}\times baseline PHQ{9}_{i}+{\beta }_{8}\times {BA}_{i}\times visi{t}_{j}+{\beta }_{9}\times {AT}_{i}\times visi{t}_{j}+{\beta }_{10}\times {PS}_{i}\times visi{t}_{j}$$

where ${Y}_{ij}$ is the outcome variable, $B{A}_{i}, {AT}_{i}$ and ${PS}_{i}$ are treatment indicators of the three components, and $visi{t}_{j}$ is a nominal variable indicating the visit time. For the within-participant covariance matrix model, we will adopt the unstructured structure. We calculated pre–post effect sizes by dividing the estimated mean changes from baseline to week 8 by the observed SD of baseline scores, and between-group effect sizes (SMD) at week 8 by dividing the estimated mean differences in change scores between groups by the observed SD of week 8 scores. No adjustment for multiple testing will be applied in the estimation of the statistical significance of the main and interaction effects in this model, and the conventional threshold for statistical significance (*p* < 0.05, two-sided) will be used because of the following: in the optimization phase of the MOST framework [42], the emphasis is on making a decision about what components will make up the optimized intervention, and factorial designs usually evaluate multiple, completely different interventions that could have been assessed in separate trials and, conventionally, the multiple hypotheses have been tested independently for these trial designs [43, 44].

#### Secondary analysis

The secondary analysis will use the same models as those used in the primary analysis. MMRM analysis will be used to estimate the mean differences in changed scores on the GAD-7 questionnaire for each component. So, we will perform the MMRM analyses involving second-order interaction effects of the three components as secondary analyses.

#### Interim analysis

We do not plan any interim analysis.

## Sample size estimation

In the smartphone psychotherapy intervention used in this study, CBT, including behavioral activation and cognitive restructuring, showed a statistically significant effect among 164 depressive patients, with an effect size of 0.3–0.4. In this study, to determine an effect size of 0.3, for each intervention factor (problem-solving skills, behavioral activation, assertive communication) and its interaction at an alpha = 0.05 and a beta = 0.20, we need a total sample size of 350. We estimated the effect size as 0.3 because problem-solving skills or behavioral activation had the same effect as full-package CBT in a recent comprehensive meta-analysis [45]. To achieve this, the sample size needs to be 22 for each of the 16 combinations, and the total sample size is therefore 16 × 22 =352.

Although we expect a dropout of approximately 20%, we will use mixed-effects repeated measures analysis.

As a study with more than five measurement points is known to require 30–50% fewer participants for the same power compared to a pre-to-post-only assessment, we set the final target sample size of our study conservatively, as estimated above.

If the case accumulation is smoother than expected, we will continue the case accumulation aiming at a sample of 787, which will enable an effect size of 0.2.

### Monitoring

#### Data monitoring

Data integrity will be monitored centrally, first through the built-in data check system on the server program and second by the data management center on a monthly basis. The data management center will prepare the monthly summary (the number of participants entering the study, the number of participants completing the study, and serious adverse events) to be presented. Since the psychological intervention provided by the application is not invasive or expected to cause serious harm, a data monitoring committee will not be organized.

#### Harm

No specific or serious adverse events are expected for the participants who use this smartphone app. However, using it might lead to psychological distress in some participants, depending on their psychological state. We evaluated these potential adverse events by qualitative evaluation of the intervention in a previous study.

#### Compensation

Our previous trials suggest that a little harm occurred in this trial. However, if any health hazards occur, they will be covered by the National Health Insurance system.

#### Auditing

Because the intervention can be classified as a “minimally invasive intervention,” no formal auditing will be conducted.

## Ethics and dissemination

### Research ethics approval

The protocol was approved by the Institutional Review Board of Nagoya City University (ID:46-20-0005) and will be approved by the ethics committees of other collaborating universities.

### Protocol amendments

If important protocol modifications are needed, the investigators will discuss and report them to the Institutional Review Board of Nagoya City University for approval. Once approved, they will be reported to all the study investigators and, when necessary, to the study participants. Further approval of the ethics committees of the participating universities will also be sought.

### Confidentiality

Each participant will receive an identification number. All records will be managed using identification numbers. The security of the data transfer between the application and the server will be guaranteed by SSL, and the data will be stored on the secure server. The data management center will download the data regularly and store them using a medium that is not connected to the Internet and is kept in a locked drawer. Once the trial is completed, the data on the server will be erased permanently. The medium storing the downloaded data will be kept in the locked drawer in the student health center for 10 years after the publication of the primary findings.

### Access to data

All members of the steering committee will have full access to the final trial dataset.

### Ancillary and post-trial care

All participants will receive the standard care, except specialized cognitive behavioral therapy, provided by the participating hospitals and corresponding facilities both throughout and after the study.

### Dissemination policy

The protocol paper and the study results will be submitted to peer-reviewed journals. The first author of the main paper will be a member of the steering committee (authors of the protocol paper). If approved by the steering committee, another person could be the first author. The list of coauthors will be determined before submitting each paper. The main and relevant findings will be presented at conferences.

## Patient and public involvement statement

The study protocol was designed with a patient (a breast cancer survivor) who participated in the study as a researcher. She appropriately discussed the protocol with other patients when a patient’s preferences and/or opinions were considered. She will play the same role in implementing the study. Therefore, patients have been and will always be included in the study. The results of the study will be made public on the study homepage.

## Discussion

We have described the protocol of the MOST fully factorial trial aimed at optimizing smartphone psychotherapy for cancer patients. The primary objective of this study is to develop the most efficient and effective smartphone psychotherapy package to address depression and anxiety in cancer patients after diagnosis to improve their quality of life. As the usability of iCBT in cancer patients has not been shown, this study will be the first to show it [46].

There will be many challenges in conducting this trial, including the recruitment of participants and their retention in the smartphone psychotherapy intervention and assessment. We have taken the following measures to address these challenges. First, we will sample patients in several high-volume cancer centers and university hospitals and ask oncologists to directly invite their patients using leaflets. Second, we have incorporated several trial design features to increase retention: the run-in period occurs before randomization so that only those who have shown preliminary adherence to the program are randomized; measuring the primary outcome at a relatively early time point (2 months); assessing all the outcomes online with several prompts built into the smartphone app; and offering appropriate monetary incentives, as approved by the ethics committee, for the completeness of data collection. In addition, we will send an e-mail to the participants who do not answer the questionnaire before the deadline. Finally, the research team has a good track record in enrolling and following participants in previous trials.

The merit of this study is that it is the first research aimed at precision medicine to determine what kind of psychotherapy, in what combination and in what order it should be provided to the vast number of cancer patients who are not receiving appropriate psychotherapy. If an effective combination of psychotherapy is determined in this study, it can be provided using smartphones to patients who cannot easily access hospitals or clinics. Considering future developments, it may be necessary to consider the appropriate targets.

We will begin recruiting in 2021 and aim to complete the trial by 2023. The results will be published soon after.

## Patient consent

Not needed.

## Trial status

This is version 1.1 of the protocol. Recruitment started on March 1, 2021, and is expected to be completed in March 2023.

## Supplementary Information

**Additional file 1.**

## Data Availability

After the publication of the primary findings, the deidentified and completely anonymized individual participant-level dataset will be posted on the UMIN-ICDR website (http://www.umin.ac.jp/icdr/index-j.html) so that it can be accessed by qualified researchers.

## Abbreviations

AT: Assertion training
BA: Behavioral activation
CBT: Cognitive behavioral therapy
EDC: electronic data capturing
e-PRO: electronic patient-reported outcome
GAD-7: Generalized Anxiety Disorder-7
ITT: Intention-to-treat
MOST: Multiphase optimization strategy
PE: Psychoeducation
PHQ-9: Patient Health Questionnaire-9
PS: Problem solving
RCT: Randomized controlled trial
SSL: Secure Socket Layer
