# Supplementary material for: Optimization of smartphone psychotherapy for depression and anxiety among patients with cancer using the multiphase optimization strategy (MOST) framework and decentralized clinical trial system (SMartphone Intervention to LEssen depression/Anxiety and GAIN resilience: SMILE AGAIN project): a protocol for a randomized controlled trial
Source: Trials. 2023 May 22;24:344. doi: 10.1186/s13063-023-07307-y (PMC10201730; doi:10.1186/s13063-023-07307-y)
Supplement: Supplementary file 1 — Additional file 1. [file 13063_2023_7307_MOESM1_ESM.pdf]

研究説明文書

SMILE AGAIN Project の研究説明文書を記載しています。

更新日:2021/01/12

本研究の実施計画と研究組織は、以下の通りです。

**研究課題名**	がん患者の抑うつ・不安に対するスマートフォン精神療法の最適化研究：革新的臨床試験システムを用いた多相最適化戦略試験		
**研究機関名**	名古屋市立大学		
**研究責任者職名・氏名**	大学院医学研究科・精神・認知・行動医学　教授　明智　龍男		
**研究分担者職名・氏名**	名古屋市立大学病院緩和ケア部	助教	内田　恵
	名古屋市立大学大学院医学	研究員	今井　文信
	名古屋市立大学看護学部	准教授	樅野　香苗
	名古屋市立大学看護学部	教授	香月　富士日
	愛知県がんセンター乳腺科	部長<br>兼副院長	岩田　広治
	国立がん研究センター東病院<br>放射線治療科	医長	全田　貞幹
	国立がん研究センター東病院<br>乳腺外科	医長	岩谷　胤生
	国立がん研究センター中央病院<br>精神腫瘍科	科長	松岡　弘道
	東北大学大学院緩和医療学	教授	井上　彰
	静岡がんセンター婦人科	医長	安部　正和
	名古屋市立大学大学院<br>医学研究科　乳腺内分泌外科	教授	遠山　竜也

共同研究者職名・氏名	名古屋市立大学大学院<br>医学研究科　乳腺内分泌外科	教授	遠山　竜也
	キャンサー・ソリューションズ<br>株式会社	代表取締役<br>社長	桜井　なおみ
	国立がん研究センター<br>支持療法開発センター	センター長	内富　庸介
	京都大学大学院<br>医学研究科健康増進・行動学分野	教授	古川　壽亮
	国立精神・神経医療研究センター<br>認知行動療法センター	センター長	堀越　勝
	東北大学大学院医学系研究科<br>医学統計学分野	教授	山口　拓洋
	国立がん研究センター中央病院・支持療法開発部門	外来研究員	益子　友恵
	同上	特任研究員	宮路　天平

1　本研究について

【本研究の意義】

皆様におかれましては、療養しながら様々な負担を抱えた日々を送っていらっしゃることと推察いたします。私たちのグループは、がんの患者さんによりよいケアを提供するための試験に取り組んでおります。現在、私たちの研究グループは、スマートフォンを用いて日常生活の困り事を解決し、活動の幅を広げ、自分の気持ちをうまく伝えることで、抑うつと不安をどれくらい和らげることができるのかを知るための試験を行っています。この支援方法は有用である可能性がありますが、まだ科学的に証明されていません。
以下に研究の内容について説明してありますので、よくお読みになった上で、ご協力いただける場合には、ホームページから参加希望の手続きにおすすみください。その後、事務局でいくつかのことを確認させていただいたうえで、ウェブサイトから研究参加への同意手続きに進ませていただきます。もし同意書類を郵送する方法での参加希望をされる場合は、事務局からの参加確認のお電話の際に郵送を希望する旨お伝えください。事務局から同意書類を郵送いたします。また、同意手続きが完了した場合、後日メールにてお知らせいたします。メールとともに、この説明同意書もダウンロードもしくは印刷して大切に保管してください。

【倫理審査について】

この研究は、公立大学法人　名古屋市立大学大学院　医学研究科長および名古屋市立大学病院長が設置する医学系研究倫理審査委員会（所在地：名古屋市瑞穂区瑞穂町字川澄１）において医学、歯学、薬学その他の医療又は臨床研究に関する専門家や専門以外の方々により倫理性や科学性が十分であるかどうかの審査を受け、実施することが承認されています。またこの委員会では、この研究が適正に実施されているか継続して審査を行います。なお、本委員会にかかわる規程等は、以下、ホームページよりご確認いただくことができます。

名古屋市立大学病院　臨床研究開発支援センター　ホームページ　“患者の皆様へ”

http://ncu-cr.jp/patient

また、本研究は名古屋市立大学大学院　医学研究科長および名古屋市立大学病院長の許可を得て実施しています。

【本研究の目的】

多くのがんの患者さんが、治療中も治療後も抑うつと不安を抱えていらっしゃることが明らかになっています。がんの患者さんの抑うつや不安に対し、日常生活の困り事を解決していくこと（これを「問題解決療法」といいます）、楽しくやりがいのある活動を生活に取り入れること（これを「行動活性化療法」といいます）、自身の気持ちを相手を傷つけたり自身を犠牲にしないで表現できるようになること（これを「アサーショントレーニング」といいます）を通して気持ちを和らげることが有効であると言われています。また、近年、スマートフォンが普及し、生活のうえで身近なものになってきていることを踏まえ、私たちのグループはスマートフォンを用いた問題解決療法、行動活性化療法アサーショントレーニングを開発いたしました。この試験は、スマートフォンを用いてこれら支援を行うことによって、どの程度、精神的な苦痛を和らげることができるかを調べます。

【研究への協力について】

１）本研究の方法

今回、研究への参加に同意をいただけましたら、全員心理教育レッスン（ストレスや健康的な心理を日々保つことの大切さに関する情報提供）に進んでいただきます。心理教育レッスンを２週間以内に終了された方のみ本研究に正式に参加したとみなされ、１６通りの精神療法の組み合わせのいずれかに割り振られます。２週間以内に心理教育レッスンを終了できなかった場合には、研究参加のご同意を自主的に撤回されたものとみなします。この割り振りに関しては、コンピューターが無作為に決定します。この決定に関しては、参加者自身あるいは研究責任者は関与することができません。この手法を「ランダム化」と言い、治療の有効性を確かめるために行われる科学的手法の一つで、１６通りのどれになるかの確率は均等にされています。

２）皆様に具体的にお願いしたいこと

①アンケート調査

研究に参加していただく皆様に対して、普段の治療やスマートフォンを用いた問題解決療法、行動活性化療法、アサーショントレーニングが行われる前後の状態を把握するために、アンケート調査を行います。これによって気持ちの状態などを把握します。皆様に調査を10回実施させて頂く予定で、時期は試験に参加されてから同意時（第0週）、第１週、第２週、第３週、第４週、第５週、第６週、第７週、第８週、第24週になります。この時期になりましたら、スマートフォンのウェブ上でアンケートにお答えください。（第4週と第8週のアンケートの入力が完了していない場合、研究事務局からメールやお電話をさせていただく予定ですのでご了承ください）。

②スマートフォンを用いた精神療法への参加

1回目、同意時（第0週）のアンケート入力後、割り付けられた精神療法をすぐに実施していただきます。スマートフォンを用いた精神療法はアプリを使い、ご自分で進めていただきます（アプリは週におおよそ３０分から４５分程度かかります。アプリはたくさん実施していただいたほうがより効果的ですので、みなさんの自習を励ますために、事務局から第8週まで、メールを毎週お送りいたします。）。割り付けられた精神療法以外の精神療法アプリに関しては、第24週のアンケートにお答えいただいた方でご希望があれば、６ヶ月（２４週間）後からアプリを使っていただきます。

【本研究の対象となる方の病状と治療と試験の流れ】

以下のすべてに当てはまる方は、その研究に参加いただけます。

1. がん診断を受けている
2. 同意取得時の年齢20歳以上
3. スマートフォンユーザー
4. 担当医が試験に参加可能な身体状態と判断した場合

また、以下の条件に1つでもあてはまる方は、この研究に参加いただけません。

1. 日本語の読み書きが難しい
2. 認知機能障害や重いうつの存在など担当医が試験への参加を不適当であると判断させていただいた場合
3. 認知行動療法を経験したことがある
4. スマートフォン認知行動療法あるいは先行研究であるスマイルプロジェクトに参加したことがある

*上記基準に関しては、臨床試験コーディネーターが最終的に電話で確認させていただきます。

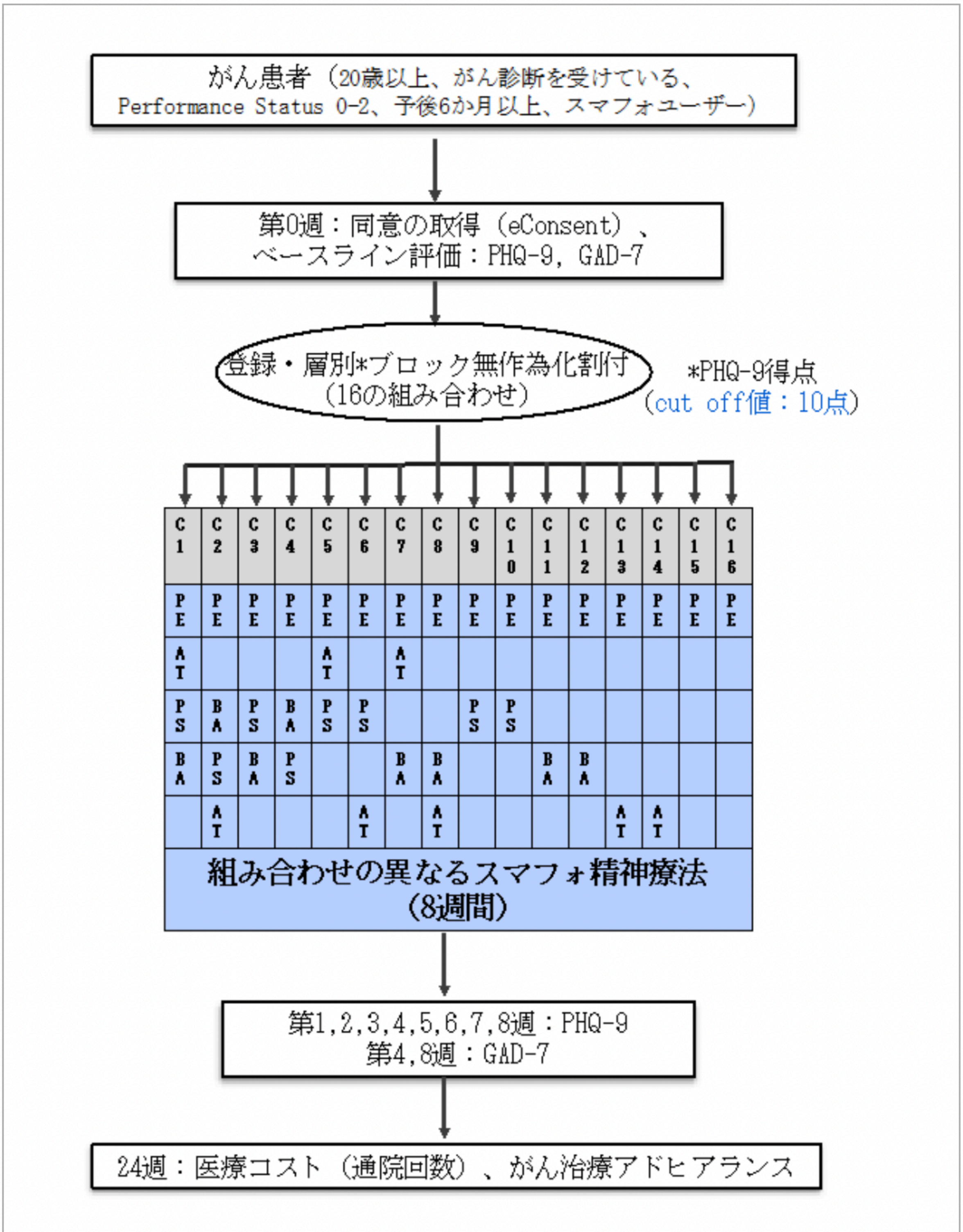

・PE (Psychoeducation　心理教育)
・BA (Behavioral activation　行動活性化療法)
・AT (Assertion training　アサーション・トレーニング)
・PS (Structured problem solving 問題解決療法)

	項目	第0週	第1週	第2週	第3週	第4週	第5週	第6週	第7週	第8週	第24週
研究者あるいは臨床試験コーディネーター	基本属性	○									
	説明と同意取得	○									
参加者	アンケート調査	◎	△	△	△	○	△	△	△	○	○

◎：10分〜15分のアンケート調査
○：5分〜10分のアンケート調査
△：数分のアンケート調査

【本研究全体の実施予定期間とあなたに参加いただく期間】

研究全体の実施予定期間は３年を予定しており、皆様に参加いただく期間は６ヶ月（２４週）です。

【本研究への参加の自由と取りやめの自由について】

本研究へのご協力は皆様個人の自由意思によるものです。いったん同意された後でも、理由を明確にすることなくいつでも同意を撤回することができます。この説明書をよまれたからといって参加されない場合や途中で撤回する場合でも、何ら不利益は生じません。同意を撤回する場合、アンケートを入力するウェブサイトに同意撤回の項目がありますので、そちらをお選びください。

2　研究に参加した場合に予想される利益および不利益・試験後の対応

本研究では、抑うつと不安に対してスマートフォンを用いた問題解決療法、行動活性化療法、アサーショントレーニングを行います。研究に参加して頂くことによって、精神心理的な負担が軽減し、生活の質が改善する可能性があります。
また、本研究は、アンケートへの入力、メール、スマートフォンのアプリ操作が主で、皆様の身体に与える悪影響はございません。しかしながら、内面的なことに触れることで皆様に不快感を与えることがあるかもしれません。一方、これまでの私どもの同様の研究の経験からは、不利益はほとんどないと考えております。万が一本試験に参加中に健康被害が生じるような状況を認めた場合は、速やかに研究事務局（090-7683-1824　平日12:00~15:00、16:00~19:00）に相談して下さるようお願いいたします。

3　健康被害が発生した場合の対応・補償について

本研究は、ほぼ危険性は伴わないため、この研究を行うことによる健康被害に対して補償や賠償保険などは準備しておりません。もし、身体状態や精神状態が悪化した場合には、お手数ですがかかりつけの医療機関でご相談いただければ幸いです。

4　研究の資金源と利益相反について

本研究は、日本医療研究開発機構の研究費「がん患者の抑うつ・不安に対するスマートフォン精神療法の最適化研究：革新的臨床試験システムを用いた多相最適化戦略試験（主任研究者　明智龍男）」、文部科学省研究費補助金「がん患者の不安、抑うつに対するスマートフォン精神療法の有効性：無作為割付比較試験（主任研究者　明智龍男）」の支援のもと行われています。

本研究における利益相反はありません。

5　個人情報の取り扱い

研究において得られたプライバシーに関する情報は厳重に守られます。あなたの名前や電話番号、メールアドレスなどの個人を識別する情報は、この研究の結果の報告や発表に使用されることはございません。

6　研究計画等の開示

皆様が希望すれば、他の参加者の個人情報や本研究の独創性の確保に支障がない範囲内で、研究計画および研究の方法に関する資料を閲覧することができます。閲覧希望の場合は事務局までお問合せください。

7　研究結果の公表

個人情報が分からないようにした上で、本研究の結果を統計学的に分析し、結果及び臨床試験を通じて得られたあなたに係わる記録が、医学および看護学の発展のため学会や学術雑誌等で公表される予定であることをご了承下さい。なお、アプリによる治療経過における治療内容、アンケートの結果などすべてが分析の対象となります。研究の進み具合やその成果については、ご希望がありましたら説明いたします。

8　研究から生ずる知的所有権について

本研究によって特許が生じた場合は、名古屋市立大学に帰属するものといたします。

9　研究期間中のデータ等について

この研究で得られたアンケート（データ）の結果は、主にスマートフォンを用いた問題解決療法、行動活性化療法、アサーショントレーニングの介入前後の状態を比較するために用います。これらのアンケートデータは、研究IDを用いて管理され、名前や年齢などの個人情報とは分けて保存するため、研究者に個人情報が知られることはございません。
また、個人情報と研究IDとの対応表については、研究事務局、受託機関（株式会社アクセライト：プライバシーマーク登録番号「17001989(02)」が閲覧できるよう別システムにて厳重に管理いたします。

10　研究終了後のデータ等について（将来の研究に用いる可能性・他の研究機関に提供する可能性）

この研究で得られたデータは、原則として研究終了後10年で、名古屋市立大学病院の機密書類として廃棄しますが、個人情報が分からないようにした上で、データを系統的レビュー及びメタアナリシスなどに二次利用することがあります。

11　本研究への参加に伴う経済的負担

スマホアプリはすべて無料でご利用いただけます。ただし、ご利用の携帯電話会社にはアプリの使用時やデータの送受信にかかる通信料はお支払いいただく必要があります。アプリを使って頂くためにパケット定額サービスの加入をお勧めします。また、本研究へのご参加に関して、些少ながら謝礼（アマゾンギフト券：全てのアンケートに回答してくださった場合は5000円分）をメールにてお送りさせていただきます。

12　連絡先

本研究に対して疑問などがある場合、ご遠慮なく事務局担当者までお問い合わせ下さい。

【問い合わせ先（平日12：00〜15：00、16：00〜19：00）】

＊問い合わせ時間以外のお時間にご連絡頂いた際には、翌平日の問い合わせ時間内にてこちらからご連絡させていただきます。

【事務局】

うちだめぐみ　いまいふみのぶ　もみのかなえ　かつきふじか　あけちたつお
内田恵、今井文信、樅野香苗、香月富士日、明智龍男

名古屋市立大学大学院医学研究科精神・認知・行動医学分野
住所：〒467-8601　名古屋市瑞穂区瑞穂町字川澄1番地
電話：090-7683-1824
メール：info@smileagain-project.org

研究参加のお申込み

研究参加のお申し込みは以下のフォームよりお願いいたします。

研究参加の申し込みをする

研究参加の申し込み

研究の参加をご希望の方は以下の入力フォームよりご登録ください。

研究参加をご希望の方は、以下の入力フォームに必要項目をご記入の上「保存して進む」ボタンをクリックしてください。
参加お申込みののち事務局よりお電話をいたします。適格性の確認と個人確認資料のご提出をお願いしております。
なお、以下のフォームの「研究参加前の確認事項」は全てを満たしている必要がございますのでご了承ください。

お名前 必須	姓 ＿＿ 名 ＿＿
お名前（ふりがな） 必須	姓 ＿＿ 名 ＿＿
ご年齢 必須	＿＿ 歳
メールアドレス 必須	※携帯電話のメールアドレスを入力される方は、予めドメイン「smileagain-project.org」からのメールを受信できる設定にしてください。Gmail アドレスをご使用の方は必ず「迷惑メール」をご確認ください。
メールアドレス（確認用） 必須	
電話番号 必須	
電話連絡 必須	**事務局から電話連絡をいたします。**<br>**ご希望の時間帯がございましたらチェックをしてください。**<br>**※ご希望の添えない可能性もございます。あらかじめご了承ください。**<br>☐ 平日 12:00～15:00<br>☐ 平日 16:00～19:00<br>☐ その他
治療のために通院されている病院名 必須	--
担当医のお名前	※わかる範囲でご入力ください。苗字のみやひらがなでの入力でも構いません。不明な場合は「不明」とご記入ください。
研究参加前の確認<br><br>【注意】<br>この項目の中に「いいえ」がひとつでもあると研究に参加できません。 必須	ホームページ上の研究説明ビデオおよび研究概要を確認した<br>○ はい　○ いいえ<br><br>申込時の年齢が 20 歳以上である<br>○ はい　○ いいえ<br><br>がんと診断されている<br>○ はい　○ いいえ<br><br>スマートフォン（iPhone/ iPad または Android）を日常的に使用している<br>○ はい　○ いいえ<br><br>担当医から試験を紹介された<br>○ はい　○ いいえ

保存して進む

PAGE TOP

Copyright 2023 SMILE AGAIN Project / Accelight Inc. All Rights Reserved.
